# Tyrosine kinase inhibitors as an alternative treatment in canine mast cell tumor

**DOI:** 10.3389/fvets.2023.1188795

**Published:** 2023-06-08

**Authors:** Yasmin Nascimento Bernardes Coelho, Luiz Ricardo Soldi, Paulo Henrique Rosa da Silva, Caio Melo Mesquita, Luiz Renato Paranhos, Thaísa Reis dos Santos, Marcelo José Barbosa Silva

**Affiliations:** ^1^Institute of Biomedical Sciences, Federal University of Uberlândia—UFU, Uberlândia, MG, Brazil; ^2^School of Dentistry, Federal University of Uberlândia—UFU, Uberlândia, MG, Brazil; ^3^School of Veterinary Medicine, Federal University of Uberlândia—UFU, Uberlândia, MG, Brazil

**Keywords:** mast cell tumor, tyrosine kinase inhibitors, vinblastine, targets therapy, dogs

## Abstract

The current gold standard treatment for canine mast cell tumors (MCT) uses vinblastine sulfate (VBL) as chemotherapy, although tyrosine kinase inhibitors (TKI) have recently been shown to be worthy candidates for treatment. This systematic review aimed to analyze the overall survival (OS), progression-free survival (PFS), overall response rate (ORR), and complete (CR) or partial response (PR) in dogs with MCT treated with TKI compared to standard VBL treatment. The systematic review was registered in the Open Science Framework (OSF) database under the identifier 10.17605/OSF.IO/WYPN4 (https://osf.io/). An electronic search was performed in nine databases. References from eligible studies were also selected to find more registers. A total of 28 studies met the eligibility criteria, and one more was recovered from the references of eligible studies, totaling 29 selected studies. The overall response rate, complete response, and partial response were higher in dogs treated with tyrosine kinase inhibitors than in dogs treated with vinblastine. The overall survival and progression-free survival of vinblastine-treated dogs were higher compared to tyrosine kinase inhibitors-treated dogs. Dogs with mutated KIT treated with tyrosine kinase inhibitors have longer overall survival and progression-free survival compared to those treated with vinblastine. It is important to consider the limitation of the study which should temper the interpretation of the results, videlicet, the extracted data lacked sample standardization and included variables such as animal characteristics, mutation detection methods, tumor characteristics, and treatment types which may have influenced the outcome of the study.

**Systematic review registration:**

https://osf.io/, identifier: 10.17605/OSF.IO/WYPN4.

## 1. Introduction

Kinases are related to tumor development due to three main carcinogenic factors: their involvement in cell proliferation, cell survival, and tumor angiogenesis (1–7). These carcinogenic factors promote uncontrolled proliferation and make tumor cells more susceptible to death (8). Kinase activation is dependent on growth factors, but when kinase mutation occurs this dependency is voided, resulting in receptor autophosphorylation and up-regulation of downstream signaling, allowing uncontrolled cell proliferation and survival (2).

The proto-oncogene c-kit encodes a receptor tyrosine kinase that plays an important role in mast cell growth and differentiation, and depending on the type of mutation in this proto-oncogene might cause ligand-independent activation of the c-kit. The incidence of mutations in the proto-oncogene c-KIT in canine mast cell tumor ranges from 14 to 26.2% and is primarily detectable in exons 8 and 11. Most mutations occur in exon 11 with an incidence of up to 64%, and ~50% of mutations are represented by internal tandem duplications (9–13). The ligand for the c-kit receptor is defined as stem cell factor (SCF), also called mast cell growth factor (MGF) (14, 15). SCF regulates the survival and proliferation of primordial germ cells and has remarkable synergistic activity in bone marrow cultures (14).

Mast cell tumors are the main skin cancer that affects dogs and have reduced survival when metastasis occurs. The collated data reveals that around 76% of affected dogs experience metastasis to regional lymph nodes, while tumor-related mortality is commonly observed in dogs exhibiting tumors graded II and grade III, with an occurrence of 25–67%, respectively (16, 17). Mast cell tumors may have a mutation in the proto-oncogene c-kit, and, therefore, the use of c-kit inhibitors for these patients is interesting. The treatment for canine mast cell tumors is chemotherapy with vinblastine (VBL) usually associated with prednisone, in addition to other drugs such as lomustine and cyclophosphamide. Other protocols cited as treatment of mast cell tumor are doxorubicin, vincristine, and cyclophosphamide (18–20). The standard chemotherapy protocol is vinblastine administered at 2 mg/m^2^ intravenously. These applications are completed weekly for 4 weeks, then bi-weekly (19). The administration of VBL has exhibited high efficacy in dogs with mast cell tumors. Evidence indicate an overall response rate corresponding to 47% in dogs with gross disease. Additionally, dogs diagnosed with grade III tumors exhibited an overall survival rate of 331 days, with 45% of subjects still alive 1–2 years after treatment (21). The range of side effects associated with this treatment encompasses a range of severities. Milder adverse effects comprise symptoms such as vomiting, lethargy, diarrhea, and neutropenia that do not culminate in sepsis. On the other hand, severe adverse effects include refractory vomiting, and severe neutropenia with fever, which may prompt the discontinuation of treatment. Neutropenia is the most significant dose-limiting toxicity of vinblastine, with an incidence of up to 73% of cases. It may culminate in a nadir of neutrophils after 1 week of treatment (21, 22).

Molecular target therapies have higher specificity for cancer cells, presenting targets in the genetic changes present in malignant cells, reducing damage to healthy tissues and improving the patient's quality of life (23). In veterinary medicine, toceranib was the first drug approved for targeted treatment in companion animals, indicated for canine mast cell tumor (23–26). It is a drug targeting multiple receptor tyrosine kinase simultaneously, including vascular endothelial growth factor receptor (VEGFR), PDGFR, c-Kit, colony-stimulating factor 1 receptor, and fms-type tyrosine kinase 3. Masitinib mesylate is another TKI approved for the treatment of canine mast cell tumor, which has inhibitory activity of c-Kit e, and other tyrosine kinase receptors, such as PDGFRs and fibroblast growth factor receptor 3 (FGFR3) (23, 27).

Evidence has demonstrated that the overall response rate resultant from the utilization of toceranib corresponds to 42.8%. These data reinforce the contention that administering TKI as standalone therapeutic agent provides an optimal therapeutic outcome and is superior to other therapeutic modalities utilized in the treatment of canine mast cell tumor. The administration of TKI can be performed on alternative days, without the necessity for intervening pauses during the therapeutic course to prevent the emergence of toxicities, as is observed in other protocols. Furthermore, the adverse effects of TKI therapy appear to be lower than those attributable to chemotherapy involving vinblastine and prednisone.

In veterinary medicine, there is limited data on the clinical efficacy of these targeted therapies in most canine neoplasms (8). Thus, the purpose of this study was to evaluate response rate, overall survival, and progression-free survival in dogs with mast cell tumors treated with tyrosine kinase inhibitors compared to standard vinblastine treatment.

## 2. Methods

### 2.1. Protocol registration, research question, and eligibility criteria

The protocol was reported according to the Preferred Reporting Items for Systematic Review and Meta-Analysis Protocols (PRISMA-P) (28) and registered in the Open Science Framework (OSF) database under number 10.17605/OSF.IO/WYPN4 (https://osf.io/). This systematic review was reported according to the preferred reporting items for Systematic Reviews and meta-analysis (PRISMA) (29, 30) and was conducted according to the Joanna Briggs Institute Manual (JBI) (31).

The review was designed to answer the following question: “Is there evidence of overall response rate, overall survival, and progression-free survival of dogs with mast cell tumors treated with tyrosine kinase inhibitors?” following the PCC strategy for structuring, in which: P (population), C (concept), and C (context): (1) Population: dogs with mast cell tumors; (2) Concept: treatment with tyrosine kinase inhibitors (3) Context: alternative treatments for dogs with mast cell tumors compared to standard treatments; (4) Case studies with five or more individuals and observational, randomized, and non-randomized studies were included; (5) There were no restrictions on publication language or year. Studies in a language other than Portuguese and English were translated using tools available online and included in the selection.

Exclusion criteria included: (1) Studies in which tyrosine kinase inhibitors were administered in dogs with other types of tumors; (2) Studies with drug treatments other than standard treatments or tyrosine kinase inhibitors; (3) Studies with variables other than the overall response rate, complete or partial response, overall survival, and progression-free survival; (3) Books, book chapters, case reports, case series with fewer than five individuals, conference papers, editorials, letters to the editor, literature reviews, and qualitative studies.

### 2.2. Sources of information, search, and selection of studies

Electronic searches were performed in the databases of BMC Veterinary, Embase, Latin American, and Caribbean Health Science Literature (LILACS), LIVIVO, and MedLine databases (via PubMed) and the Web of Science citation database. The EASY, Google Scholar, and Open Access Thesis and Dissertations (OATD) were used to partially capture the “gray literature.” These strategies were implemented to minimize selection publication bias. The MedLine search was constantly updated with electronic alerts, until June 2022. The search descriptors were selected according to the resources of MeSH (Medical Subject Headings), DCS (Health Sciences Descriptors), and Emtree (Embase Subject Headings). Several combinations among the descriptors were performed with the Boolean operators “AND” and “OR,” respecting the syntax rules of each database. Table 1 shows more details of search strategies and databases.

**Table 1:** Strategies for database search.

**Database**	**Search strategy (June 22, 2022)**
**Main databases**	
BMC Veterinary https://bmcvetres.biomedcentral.com/	((“Dogs” OR “Canine”) AND (“Mastocytoma” OR “Mast Cell Tumor”) AND (“Chemotherapy” OR “Tyrosine Kinase Inhibitors” OR “Toceranib” OR “Palladia” OR “Masitinib” OR “Imatinib” OR “Vinblastine”))
Embase https://www.embase.com/	(“dogs”/exp OR “dogs” OR “canine”/exp OR “canine”) AND (“mastocytoma”/exp OR “mastocytoma” OR “mast cell tumor”/exp OR “mast cell tumor”) AND (“chemotherapy”/exp OR “chemotherapy” OR “tyrosine kinase inhibitors” OR “toceranib”/exp OR “toceranib” OR “palladia”/exp OR “palladia” OR “masitinib”/exp OR “masitinib” OR “imatinib”/exp OR “imatinib” OR “vinblastine”/exp OR “vinblastine”)
LILACS https://lilacs.bvsalud.org/en/	(“dogs” OR “canine”) AND (“mastocytoma” OR “mast cell tumor”) AND (“chemotherapy” OR “tyrosine kinase inhibitors” OR “toceranib” OR “palladia” OR “masitinib” OR “imatinib” OR “vinblastine”) AND (db:(“LILACS”))
LIVIVO https://www.livivo.de/	#1 (“Dogs” OR “Canine”) #2 (“Mastocytoma” OR “Mast Cell Tumor”) #3 (“Chemotherapy” OR “Tyrosine Kinase Inhibitors” OR “Toceranib” OR “Palladia” OR “Masitinib” OR “Imatinib” OR “Vinblastine”)
	#1 AND #2 AND #3
MEDLINE (via PubMed) http://www.ncbi.nlm.nih.gov/pubmed	#1 “Dogs”[Mesh] OR “Canine”[tw] #2 “Mastocytoma”[Mesh] OR “Mast Cell Tumor”[tw] #3 “Drug Therapy”[Mesh] OR “Chemotherapy”[tw] OR “Toceranib”[tw] OR “Masitinib”[tw] OR “Imatinib”[tw] OR “Vinblastine”[Mesh]
	#1 AND #2 AND #3
Web of Science http://apps.webofknowledge.com/	#1 TS=(“Dogs” OR “Canine”) #2 TS=(“Mastocytoma” OR “Mast Cell Tumor”) #3 TS=(“Chemotherapy” OR “Tyrosine Kinase Inhibitors” OR “Toceranib” OR “Palladia” OR “Masitinib” OR “Imatinib” OR “Vinblastine”)
	#1 AND #2 AND #3
**Gray literature**	
EASY https://easy.dans.knaw.nl/	((“Dogs” OR “Canine”) AND (“Mastocytoma” OR “Mast Cell Tumor”))
Google Scholar https://scholar.google.com.br/	((“dogs” OR “canine”) AND (“mast cell tumor” OR “mastocytoma”) AND (“tyrosine kinase inhibitors” OR “toceranib” OR “palladia” OR “masitinib” OR “imatinib” OR “chemotherapy” OR “vinblastine”)) filetype:pdf
OATD https://oatd.org/	((“Dogs” OR “Canine”) AND (“Mastocytoma” OR “Mast Cell Tumor”) AND (“Chemotherapy” OR “Tyrosine Kinase Inhibitors” OR “Toceranib” OR “Palladia” OR “Masitinib” OR “Imatinib” OR “Vinblastine”))

The results obtained were exported to EndNote Web^TM^ software (Clarivate^TM^ Analytics, Philadelphia, USA), where duplicates were automatically removed, and the remaining duplicates were manually removed. The remaining results were exported to Rayyan QCRI (Qatar Computing Research Institute, Doha, Qatar) for the study selection phase. The gray literature was manually analyzed, simultaneously and fully, with Microsoft Word^TM^ 2010 (Microsoft Ltd., Washington, USA).

Before selecting the studies, two reviewers completed a calibration exercise, in which they discussed eligibility criteria and applied them to a sample of 20% of the retrieved studies to determine interexaminer agreement. After reaching an adequate level of agreement (Kappa < 0.81), the selection started.

In the first phase, two eligibility reviewers (YNBC and LRS) methodically analyzed the titles and abstracts of the studies independently. Disagreements between examiners were analyzed and defined by a third examiner (PHRS). This phase excluded titles that were unrelated to the topic, as well as abstracts that did not meet the eligibility criteria. In the second phase, the full texts of the preliminary eligible studies were obtained and evaluated. The references of eligible studies were also selected to search for more registers. If the full texts could not be located, a bibliographic request was made to the library database (COMUT) and an e-mail was sent to the corresponding authors in order to retrieve the texts.

### 2.3. Sponsorship status evaluation

The source of funding of the extracted studies was evaluated. These data are important because studies sponsored by the pharmaceutical industry are more often favorable to the sponsor's product compared to studies with other sources of sponsorship, and are related to risk of bias (32). The sponsorship status was classified as follows (33): Unclear: Studies that did not present sponsorship statements, and it is not possible to state whether they were sponsored; Non-sponsored: studies that the authors stated that there was no financial support from pharmaceutical industries and Sponsored: studies that the authors declared any kind of financial support (financial support, provision of equipment or supplies, discounts, etc.) from pharmaceutical industries.

## 3. Results

### 3.1. Study selection

The electronic search identified 1.555 results distributed in nine electronic databases, including the “gray literature.” After removing the duplicates, 1.144 results remained for the analysis. A careful reading of the titles and abstracts excluded 1.098 results.

**Figure 1:**
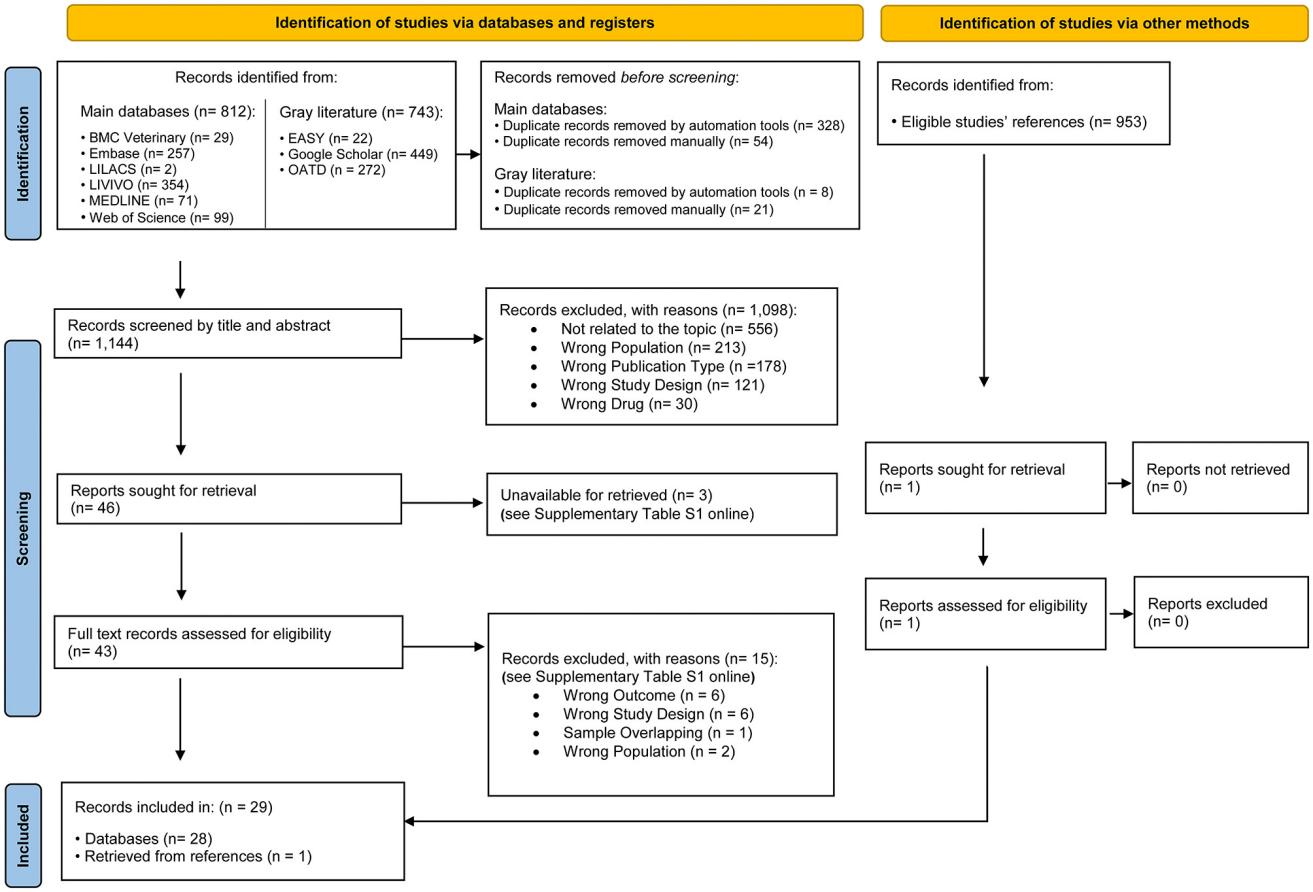
Flowchart of the systematic literature review process. From: Page et al. (32). For more information, visit: http://www.prisma-statement.org/.

After reading the full texts, 15 studies with reasoning were excluded, and 3 studies were unavailable for retrieval (see Supplementary Table 1 online). Thus, 28 studies remained in the qualitative analysis (21, 34–59). Furthermore, it examined the references of publications eligible for the recovery of studies; just one study of 953 papers examined was determined eligible and considered in this review (60). Figure 1 displays details of the study selection process.

### 3.2. Study characteristics

The studies were published from 1999 to 2022 and conducted in nine different countries, with 17 studies in North America (21, 34, 36, 37, 39, 40, 42, 44–46, 48–50, 55, 57, 58, 61), four in Europe (38, 51–53), three in Oceania (35, 43, 47, 56), two in Asia (41, 60), and two in South America (54, 59). Among the 29 eligible studies, 12 were randomized (34, 39, 47, 48, 54, 55, 57, 58), and 17 were non-randomized (21, 35–38, 41–45, 49–53, 56, 59). The sum of participants in the eligible studies resulted in 1.556 patients.

Three types of tyrosine kinase inhibitors were identified: imatinib, toceranib, and masitinib. Six studies used these drugs alone (41, 45, 50–52, 54) and four studies associated them with other chemotherapy drugs (lomustine, vinblastine, and cyclophosphamide) (46, 48, 56, 58). Eight studies compared them to vinblastine or placebo (34, 39, 44, 47, 53, 55, 57, 59), seven studies evaluated vinblastine alone (21, 35, 36, 38, 42, 60, 61), and four studies evaluated vinblastine associated with other chemotherapy drugs (37, 40, 43, 49). Regarding the treatment regimen, 609 dogs used the treatments as first-line treatment, and 563 dogs used them as adjuvant treatment. Table 2 presents the main characteristics of each eligible study.

**Table 2:** Main characteristics of eligible studies.

**References (country)**	**Study design**	**Sample**	**Drug treatment performed**	**Treatment performed as (*n*)**	Histological grade^**^(*n*)	**c-KIT mutation (*n*)**
Thamm et al. (21) (USA)	nRCT	41	VBL^*^	First-line: 28 Adjuvant: 13	Grade II: 18 Grade III: 23	nr
David et al. (53) (Australia)	nRCT	27	VBL^*^	Adjuvant: 27	High-grade: 03 Intermediate-grade: 24	nr
Thamm et al. (54) (USA)	nRCT	61	VBL^*^	First-line: 57 Adjuvant: 04	Grade II: 47 Grade III: 14	nr
Camps-Palau et al. (55) (USA)	nRCT	35	VBL^*^+ CTX	First-line: 20 Adjuvant: 15	Grade II: 26 Grade III: 09	nr
Hayes et al. (56) (United Kingdom)	nRCT	14	VBL^*^	Adjuvant: 14	Grade III: 14	nr
Hahn et al. (57) (USA)	RCT	202	T/PCB	First-line: 85 Adjuvant: 117	Grade II: 173 Grade III: 29	nr
Isotani et al. (59) (Japan)	nRCT	21	I	First-line: 10 Adjuvant: 11	Grade II: 07 Grade III: 03 nd: 11	Mutated: 05 Wild—type: 16
Rassnick et al. (61) (USA)	RCT	51	VBL	First-line: 30 Adjuvant: 21	Grade II: 24 Grade III: 26 nd: 01	nr
Rungsipipat et al. (60) (Thailand)	RCT	23	VBL	nr	Grade II: 23	nr
Webster et al. (36) (USA)	nRCT	28	VBL^*^	Adjuvant: 28	Grade II: 10 Grade III: 18	Mutated: 04 Wild—type: 24
Cooper et al. (37) (Australia)	nRCT	56	CCNU + VBL^*^	First-line: nr Adjuvant: 16	Grade II: 12 Grade III: 17 nd: 08	nr
London et al. (28) (USA)	RCT	149	T/PCB	First-line: 83 Adjuvant: 66	nr	Mutated: 11
Hahn et al. (38) (USA)	RCT	132	M/PCB	nr	Grade II: 121 Grade III: 11	Mutated: 38
Rassnick et al. (58) (USA)	nRCT	52	CCNU + VBL^*^	First-line: 17 Adjuvant: 35	Grade II: 23 Grade III: 23 nd: 06	nr
Carlsten et al. (39) (USA)	nRCT	17	T^*^	nr	Grade I: 1 Grade III: 04 nd: 12	Mutated: 06 Wild—type: 08
Robat et al. (40) (USA)	RCT	14	T + VBL	First-line: 13 Adjuvant: 05	Grade II: 07 Grade III: 05 nd: 02	nr
O'Connell et al. (41) (Australia)	RCT	63	T/VBL	Adjuvant: 63	Grade I/II: 04 Grade III: 09	nr
Burton et al. (42) (USA)	RCT	41	T + CCNU	First-line: 02 Adjuvant: 39	nr	Mutated: 15 Wild—type: 23
Lejeune et al. (43) (USA)	nRCT	25	CCNU + VBL^*^	Adjuvant: 21	nr	Mutated: 11
Smrkovski et al. (44) (USA)	nRCT	26	M/VBL	First-line: 14 Adjuvant: 12	Grade II: 09 Grade III: 17	nr
Chocteau et al. (45) (France)	nRCT	96	T	nr	High-grade: 53 Low-grade: 43	Mutated: 29 Wild—type: 67
Grant et al. (46) (United Kingdom)	nRCT	39	M	First-line: 31 Adjuvant: 8	Grade II: 19 Grade III: 10 nd: 10	nr
Miller et al. (47) (United Kingdom)	nRCT	94	M/VBL^*^	First-line: 42 Adjuvant: 52	Grade II: 45 Grade III: 44	nr
Horta et al. (49) (Brazil)	RCT	24	T	First-line: 11 Adjuvant: 13	Grade II: 13 Grade III: 11	Mutated: 6 Wild—type: 18
Moirano et al. (49) (USA)	nRCT	40	T/VBL	First-line: 40	Grade II: 13 Grade III: 09	nr
Olsen et al. (50) (Australia)	RCT	40	T +VBL	Neoadjuvant: 16 Adjuvant: 11 Palliative: 13	Grade III/High: 10 Grade II/High: 11 Grade II/Low: 19	nr
Weishaar et al. (51) (USA)	RCT	88	T/VBL	First-line: 88	Grade II: 70 Grade III: 14	Mutated: 20 Wild—type: 67
Todd et al. (52) (Canada)	nRCT	28	T + VBL	nr	High-grade: 19 Low-grade: 02	nr
Macedo et al. (29) (Brazil)	RCT	29	I/VBL	nr	Grade I: 10 Grade II: 25 Grade III: 3	Mutated: 04 Wild—type: 25

nr, not related on the study; nRCT, non-randomized clinical trials; RCT, randomized controlled trial; VBL, Vinblastine; VBL^*^, Vinblastine in combination with prednisone; T, Toceranib; M, Masitinib; I, Imatinib; PCB, placebo; T^*^, associated with hypofractionated radiation treatment and prednisolone; CTX, Cyclophosphamide; CCNU, Lomustine; nd, not determined.

^**^Patnaik et al. (62) and Kiupel et al. (63).

In order to achieve an appropriate and comprehensive comparative analysis, a total of four distinct cohorts were established: (1) the VBL group, which was treated exclusively with vinblastine chemotherapy; (2) the TKI group, which was solely administered a tyrosine kinase inhibitor; (3) the Chemotherapy + TKI group, which received either vinblastine or other forms of chemotherapy in conjunction with a tyrosine kinase inhibitor adjuvant; (4) the Other + VBL group, which was subjected to vinblastine chemotherapy accompanied by a non-tyrosine kinase inhibitor adjuvant (such as prednisone, for example). These groups were also juxtaposed with surgical interventions and assessments of metastatic risk.

### 3.3. Individual results of the studies

In the TKI group included in the present analysis, outcomes were classified as positive, neutral, and negative, with each classification being dependent on the principal objectives and hypotheses of the respective investigations. The positive results were defined as those studies which demonstrated supportive evidence for the employment of any of the drugs investigated across the four groups, and which effectively proved the efficacy of the treatments in the management of mast cell tumors. The neutral results indicated no discernible differences when comparing the efficacy of one or more drugs evaluated in each study, and there is no scientific support to claim that one drug is more effective than the other. The negative results are those outcomes that contradicted the hypotheses of the investigation, indicating that the main drug being evaluated within the four groups was inferior to another form of therapy. The main findings of the selected studies are presented in Table 3.

**Table 3:** Main outcomes of eligible studies.

**References**	**Main outcomes**
Thamm et al. (21)	Prednisone and VBL provided longer survival in patients with grade III MCTs than have surgery alone. Overall survival rate for the entire patient population was not reached with a median follow-up of 573 days; however, the OS for dogs with grade III MCT was 331 days. Overall response rate in the evaluable dogs with gross disease was 47% (7/15), consisting of five complete responses and two partial responses.
Davies et al. (53)	In seven dogs given adjunctive chemotherapy, disease-free overall survivals were considered improved over expected results with surgery alone. The protocol reported was well-tolerated by most animals.
Thamm et al. (54)	Prednisone and VBL chemotherapy are well-tolerated, and results in good outcomes following surgery in dogs with MCT at high risk for metastasis.
Camps-Palau et al. (55)^*^	The VCP protocol should be considered as an option in the treatment of MCT in dogs. The median progression-free survival for measurable disease (group 1) and excised MCT or were at high risk for metastasis (group 2) was 74 and 865 days, respectively. The median overall survival rate for group 1 and 2 dogs was 145 and >2,092 days, respectively.
Hayes et al. (56)	The VBL and prednisolone chemotherapy are a well-tolerated adjunct to surgery for grade III mast cell tumors and appears to prolong survival compared with that expected with surgery alone.
Hahn et al. (57)^*^	Masitinib is safe and effective at delaying tumor progression in dogs presenting with recurrent or non-resectable grade II or III non-metastatic MCT.
Isononi et al. (59)	Imatinib mesylate has clinical activity against MCT in dogs. Ten of 21 dogs (48%) had some beneficial response to imatinib mesylate treatment within 14 days of treatment initiation.
Rassnick et al. (61)^*^	VBL, when used as a single-agent, has activity against MCTs in dogs although the response rate is lower than those reported for VBL-containing combination protocols. In the VBL 2.0 group (2.0 mg/m^2^), 3 (12%) had a partial response for a median of 77 days (range, 48–229 days). Overall response rate in the VBL 3.5 (3.5 mg/m^2^) group was 27%. One dog (4%) had a complete response for 63 days and 6 dogs (23%) had a partial response for a median of 28 days (range, 28–78 days).
Rungsipipat et al. (60)	Group 1 treated with VBL and prednisolone had a shorter overall survival (101 days) than those treated with prednisolone only (175 days). With regard to the clinical evaluation, 18 dogs (78.2%) were partially responsive and the rest (21.8%) were stable in group 1 while five dogs (50%) were partially responsive, three dogs (30%) were stable, and the remaining two dogs (20%) were progressive in group 2.
Webster et al. (36)^*^	Treatment with VBL and prednisone following surgery +/– RT benefited dogs with grade III MCT over treatment with surgery alone.
Cooper et al. (37)	Evaluating the protocol CCNU and VBL, 57% response rate was seen in dogs with macroscopic disease for a median duration of 52 weeks. Dogs with macroscopic disease had a median progression free overall survival of 30 weeks and a median overall survival of 35 weeks.
London et al. (24)^*^	This study provides the first evidence that administered kinase inhibitors can exhibit activity against a variety of spontaneous malignancies.
Hahn et al. (38)^*^	Masitinib significantly increased survival rates at 12 and 24 months in dogs with non-resectable MCTs.
Rassnick et al. (58)^*^	The PFS time in dogs treated in the adjuvant setting was 489 days. Dogs with grade III MCTs had shorter PFS compared with dogs with metastatic grade II MCTs (190 vs. 954 days; *P* < 0.001).
Carlsten et al. (39)	The combination of hypo fractionated RT, toceranib, and prednisone was tolerated and efficacious in most dogs. Response rates and durations were higher than those reported for toceranib as a single-agent treatment for MCT.
Robat et al. (40)^*^	The combination of VBL and TOC does appear to have significant activity and is generally well-tolerated and there is a suggestion of additive or synergistic activity when the agents are combined. The complete response occurred in two dogs (14%) and partial response was observed in eight dogs (57%) for a best overall observed response rate of 71%.
O'Connell et al. (41)	No significant difference in survival was found for dogs receiving therapy with VBL alone.
Burton et al. (42)	Combined treatment with pulse-administered TOC and CCNU generally is well-tolerated and may be a reasonable treatment option for dogs with unresectable or metastatic MCT. The overall response rate was 46% (four complete response, 15 partial response) and the overall median progression-free survival was 53 days (1 to >752 days).
Lejeune et al. (43)	The use of prednisone, VBL and CCNU after adequate local-regional therapy can provide an overall survival rate more than 40 months. The overall survival rate for all dogs was 1,359 days (range, 188–2,340). Median disease-free interval was 2,120 days (149–2,325 days)
Smrkovski et al. (44)^*^	The overall response rate to masitinib was 50%. The median overall survival for dogs that responded to masitinib was 630 vs. 137 days for dogs that did not respond.
Chocteau et al. (45)^*^	TKI treatment significantly increased the mean overall survival of dogs bearing c-Kit-mutated but had a deleterious effect on survival of dogs with wild-type.
Grant et al. (46)	Masitinib appears to be a well-tolerated and effective drug against macroscopic mast cell tumors. Clinical response was observed in 32 (82.1%) dogs receiving masitinib, with 15 dogs (38.5%) exhibiting a complete response and 17 dogs (43.6%) achieving a partial response. The median time to progression was 79 days (range: 14–667 days).
Miller et al. (47)^*^	Patients with a surgically excised Patnaik grade II tumor and high Ki-67 in the absence of metastatic disease treated with VBL and prednisolone showed a significantly longer survival (MST: 1,946 days) than those treated with masitinib (MST: 369 days).
Horta et al. (48)^*^	A total of 12/24 dogs achieved an overall response and the overall survival for all subjects was 113 days. Dogs responding to treatment had a significant increase in overall survival compared to non-responders (146.5 vs. 47 days, *P* = 0.02).
Moirando et al. (49)^*^	The combination of CCNU, VBL and prednisone prolonging survival over the TOC.
Olsen et al. (50)	The combination of VBL, prednisolone and TOC demonstrated response in 90% (26/29) patients with measurable disease. The overall survival rate for patients who received adjuvant therapy following surgical resection was 893 and 218 days, for patients being palliated for gross metastatic disease.
Weishaar et al. (51)	Neither PFS or OS was significantly different between treatment group (TOC and VBL). As the proportion of dogs with c-kit mutations was not different between treatment groups in this population of dogs, c-kit mutation status did not predict treatment response.
Todd et al. (52)	The combination VBL and TOC was well-tolerated. Progression-free intervals and overall survivals were similar in dogs with high-grade, metastatic and Stage IV disease.
Macedo et al. (29)^*^	The objective response rate (ORR) was significantly higher (30.79%) in the imatinib mesylate group then in vinblastine and prednisone group (9.09%). The imatinib mesylate group presents some advantages compared to conventional chemotherapy and may be used for the benefit and comfort of dogs with low-grade MCT.

^*^Studies that presented significant data.

VBL, Vimblastine; MCT, mast cell tumor; VCP, protocol of vinblastine, cyclophosphamide, and prednisone; RT, radiotherapy; CCNU, Lomustine; PFS, progression-free survival; TOC, Toceranib; ORR, overall response rate; TKI, tyrosine kinase inhibitors; OS, overall survival.

Positive results were observed in 21 articles. The VBL group had better results when compared to surgery alone, and also presented effectiveness as an adjunct to surgery (21, 35, 36, 38, 42). TKI has been shown to have positive effects, with included publications highlighting that they are safe, effective, and have clinical activity against mast cell tumors in dogs (34, 39, 41, 44, 50–52, 59, 61). In the Other + VBL group, cyclophosphamide and lomustine proved to be well-tolerated and showed efficacy concerning treatment response and overall survival (37, 40, 43, 49). The Chemotherapy + TKI group showed good tolerance and positive results concerning response to treatment and overall survival, which can be justified by the additive and synergistic action of the combination of these agents (45, 46, 48, 56, 58).

Regarding the positive results, 28.5% (*n* = 6) of the studies had sponsorship declarations by the drug manufacturers or with authors associated with an industry: three received financial support from Pfizer Animal Health (34, 45, 46), two received sponsorship from AB Science, S.A. (39, 44), and one received financial support from Zoetis, Inc. (48). About 57% (*n* = 12) of the studies did not present a sponsorship declaration and could not state whether there was financial support or not, and in only 19.0% (*n* = 4) studies the authors declared that there was no conflict of interest.

Two studies presented neutral results that showed no difference in the progression-free interval, overall-survival, complete response, objective response rate, or clinical benefit between VBL treatment and TKI (47, 57). Furthermore, there was no difference in outcome in the intermediate-risk for metastasis group treated with toceranib in one study (54).

Concerning the negative results, the efficacy of treatment in the VBL and Other + VBL groups was superior compared to the TKI group. However, TKIs were still well-tolerated and showed effectiveness in the treatment of mast cell tumors (53, 55). Another negative outcome was seen in the VBL group and in the Other + VBL (combination with prednisolone), where a shorter overall survival was found when compared to dogs that were treated with prednisolone only (60).

It is important to note that not all studies yielded statistical analysis. Certain studies solely evaluated the efficacy of drugs without placebo control and therefore did not provide sufficient data to permit inter-group comparisons. Similarly, other studies comparing two groups may have lacked the needed statistical data required to perform statistical analysis.

### 3.4. Data synthesis

The results were collected from the overall response rate, complete or partial response, overall survival, and progression-free survival in the groups of the eligible studies. The variables extracted depended on the type of study collected, such as the presence of groups according to the characteristics of the animal, the treatment, and the tumor. If a study presented totals of the variables evaluated, without group separations, these data were extracted in total from all animals, without considering other characteristics of the study sample. However, if the study presented data separated into groups according to the characteristics of each patient (such as the presence of c-kit mutation, histological grade, type of treatment, and tumor measurement), a mean was performed to obtain the total value. The values extracted individually from each group evaluated in the studies were also presented. Supplementary Tables 2–5 online summarized these quantitative data.

To evidence the different methods of detection of mutation of the oncogene c-kit that may present variation in its sensitivity, the main methodologies of detection of each study that evaluated the mutation status were described, as well as the classifications of the mutation type (Internal Tandem Duplications or Point Mutation) and the amplified and sequenced exons. These data are described in the Supplementary Table 6.

### 3.5. Industry sponsorship status

The sponsorship status is shown in Table 4. Seven studies declared financial support or presented authors associated with pharmaceutical industries that manufactured the drugs evaluated. In five studies, the authors declared no conflicts of interest. Unclear information was observed in 17 studies, and it was not possible to affirm the sponsorship status of the study.

**Table 4:** Sponsorship status of the studies.

**References**	**Sponsorship status**	**Sponsorship**
Thamm et al. (21)	Unclear	
Davies et al. (53)	Unclear	
Thamm et al. (54)	Unclear	
Camps-Palau et al. (55)	Unclear	
Hayes et al. (56)	Unclear	
Hanh et al. (57)	Sponsored	AB Science, S.A
Isotoni et al. (59)	Unclear	
Rassnick et al. (61)	Unclear	
Rungsipipat et al. (60)	Unclear	
Webster et al. (36)	Unclear	
Cooper et al. (37)	Non-sponsored	“There was no external financial support for this study.”
London et al. (28)	Sponsored	Pfizer Animal Health Six authors are consultants of Pfizer.
Hahn et al. (38)	Sponsored	AB Science, S.A Two authors work for pharmaceutical company sponsor.
Rassnick et al. (58)	Unclear	
Carlsten et al. (39)	Sponsored	Pfizer Animal Health
Robat et al. (40)	Sponsored	Pfizer Animal Health
O'Connell et al. (41)	Unclear	
Smrkovski et al. (44)	Unclear	
Burton et al. (42)	Sponsored	Zoetis, Inc.
Lejeune et al. (43)	Unclear	
Chocteau et al. (45)	Unclear	
Grant et al. (46)	Non-sponsored	“None of the authors of this article has a financial or personal relationship with other people or organizations that could inappropriately influence or bias the content of the paper.”
Miller et al. (47)	Non-sponsored	“Authors disclose no conflict of interest.”
Horta et al. (48)	Unclear	
Moirando et al. (49)	Unclear	
Olsen et al. (50)	Non-sponsored	“The authors do not disclose any conflicts of interest.”
Weishaar et al. (51)	Sponsored	Zoetis, Inc.
Todd et al. (52)	Unclear	
Macedo et al. (29)	Non-sponsored	“The authors declare that they have no conflict of interest”

### 3.6. General characteristics

The mean follow-up of treatment responses was 785 days. In studies that had information on previous treatments, 51.9% of the animals received the tyrosine kinase inhibitor or vinblastine as the first line of treatment, while 48.03% received previous treatments. In studies that specified the type of previous treatment, it was observed that 28.5% received some type of surgical excision, 39.1% received chemotherapy, and 32.2% received radiation therapy.

In dogs that received TKIs, in studies provide information, 257 dogs received treatment under the label, and 261 received the drug off-label: 87 dogs received Masitinib for presenting non-resectable mast cell tumors (Grade II or III) with confirmed mutated c-kit tyrosine kinase. No information was collected from three studies, as they did not present the mutation status of the c-kit; 170 dogs received Toceranib because they presented mast cell tumors with histological classification in Patnaik grade II or III, recurrent, with or without regional lymph node involvement. In one study only four patients underwent the histopathological examination, so it is undetermined whether other dogs received the drug under label. All 34 animals that received Imatinib were disregarded, considering that this drug is not labeled for dogs; therefore, the use of this drug in the dog is off-label.

Among the 1.134 dogs included in the studies with reported gender, 499 (44%) were males and 635 (55.9%) were females. The mean age of the dogs in the selected studies was 9.1 years (7.6 ⊥ 16.6).

Only 1,058 dogs had histological grades following the criteria of Patnaik (62). Most dogs had grade II tumors (*n* = 714, 67.4%), followed by grade III (*n* = 328, 31%), grade I (*n* = 16, 1.5%), and lastly undetermined tumors (*n* = 24, 2.29%). This information was presented in 25 studies. In other publications, the histological degree was performed using the criteria of Kiupel (63) in which 50.5% (*n* = 133) were classified as high-grade malignancy and 136 (49.4%) with intermediate/low-grade malignancy. However, these criteria were only evaluated in nine studies, and 55.02% of tumors were classified as microscopic diseases and 44.7% as macroscopic diseases.

Regarding c-kit, 29.7% (*n* = 188) dogs presented tumors with a c-kit mutation and 71.2% (*n* = 466) had wild-type c-kit. In the studies that presented mutation prevalence data in exons, about 67.5% (*n* = 77) had mutations in exon 11, and 32.4% (*n* = 37) had a mutation in exon 8. Regarding the assignment of the standard kit, 16.2% expressed the kit type I pattern (perimembranous expression), 53.8% expressed standard kit II (focal cytoplasmic marking) and 29.9% had kit III patterns (diffuse cytoplasmically).

Concerning the type of detection methodology, these mutations were performed by reverse transcriptase-PCR assay performed on biopsy, and immunohistochemical for the evaluation of KIT protein localization. Most of the mutant tumors evaluated in the studies were based on ITD and sequenced mutations in exon 11 or 8. Only one study sequenced rarer exons such as 8–13 and 17–19. The methodology of each article and the variation of mutation detection are described in the Supplementary Table 6.

### 3.7. Overall response rate

In the TKI group, the mean overall response rate (ORR) value was 50.36% (138/274) (34, 41, 45, 50, 52, 54, 57, 59). In the VBL group, the mean ORR was 28.75% (23/80) (21, 57, 59, 61). In the Chemotherapy + TKI group (combination with CCNU or vinblastine), ORR was 65.47% (55/84) (46, 48, 56). In the Other + VBL group (associated with lomustine), the ORR was 57.53% (42/73) (40, 43). The data presented are described in Supplementary Table 7.

### 3.8. Complete and partial response

The complete response (CR) of the TKI group value reached 26.49% (133/502) (34, 39, 41, 44, 45, 50, 52, 54, 57, 59). The CR of the VBL group reached 23.21% (26/112) (21, 35, 40, 57, 59–61). In the Chemotherapy + TKI group (association with VBL or CCNU) the CR reached 33.68% (32/95) (46, 48, 56). The Other + VBL group (association with cyclophosphamide and CCNU) presented a CR of 29.23% (19/65) (37, 40, 43).

In the TKI group, the PR achieved was 39.46% (148/375) (34, 39, 41, 45, 50, 52, 54, 59). However, the PR of the VBL group reached a percentage of 36% (27/75). In the Chemotherapy + TKI group (association with CCNU or VBL), the PR found was 29.1% (16/55) (46, 48), and the Other + VBL (association with CTX or CCNU) reached a PR of 30.7% (20/65) (37, 40, 43, 59). The data presented are described in Supplementary Table 7.

### 3.9. Overall survival

Data on overall survival collected from all included studies were analyzed as follows: mean survival (mean of all included studies within the specific treatment group), mean survival according to tumor grading (I, II, III, or Low/High) (62, 63), and according to the presence of c-kit mutations (mutated or not).

In the TKI group, the mean overall survival of all dogs was 308 days (113 ⊥ 1.018). The mean overall survival in dogs with grade II mast cell tumors within this group was greater (369 days) when compared to grade III (278 days) (53). In a study organized by grade, high-grade mast cell tumors had an overall survival of 432 days (51). The overall survival of dogs with mutated KIT was 461 days, and in dogs without the mutation, the overall survival was 1.389 days (39, 51).

In the VBL group, the mean overall survival of the included studies was 524 days (101 ⊥ 1.374) (21, 36, 42, 53, 55–57, 60). In this group, mast cell tumors of grade II had a mean overall survival of 1.300 days and in grade, III had a mean of 234 days. Dogs with mutated c-kit had an overall survival of 270 days and those not mutated were 529 days (42).

The dogs in the Chemotherapy + TKI group that used VBL as a chemotherapy agent for adjuvant treatment had an overall survival of 893 days, and dogs undergoing palliative treatment survived for 218 days (total mean: 555 days). Dogs with high-grade mast cell tumors had an overall survival of 563 days. The total mean obtained from these dogs was 559 days (555 ⊥ 563) (56, 58).

In the Other + VBL group, the mean overall survival was 728 days (209.5 ⊥ 1359). The dogs with measurable disease had a mean overall survival of 195 days and those with a non-measurable disease had a mean of 1.214 days (37, 43, 49). The data presented are described in Supplementary Table 8.

### 3.10. Progression-free survival

Progressive survival was collected from all included studies and was analyzed as follows: Mean progression-free survival (mean of all included studies within a specific treatment group), mean progression-free survival according to the line of therapies (first line or second line), type of chemotherapy treatment (adjuvant or palliative), tumor classification (I, II, III, or Low/High) (62, 63), dogs with mensurable disease or dogs with incompletely excised tumor, and according to the presence of c-kit mutations (mutated or not).

In the TKI group, it was possible to obtain a progression-free interval with a mean of 207 days (30 ⊥ 453) (39, 45, 47, 50, 52, 54). Masitinib, when given as the first line of treatment, the mean PFS was 253 days (52). The PFS of dogs treated with masitinib in the second line and beyond was 84 days, dogs with mutate kit presented a mean of 209 days, and those with not mutated kit reached 72 days (39, 45). The mean PFS of the VBL group was 714 days (45 ⊥ 1.305) (36, 38, 47, 57).

The Chemotherapy + TKI group had a mean of 49 days (45 ⊥ 53) (45 days for palliative treatment; toceranib associated with CCNU and vinblastine) (48, 56). For other + VBL (combination with cyclophosphamide and CCNU), the PFS was 861 days (227 ⊥ 2.120). In grade II tumors, the PFS was 954 days, and for grade III, 190 days (37, 40, 43, 49). Dogs with measurable disease reached a mean of 142 days and dogs with incompletely excised tumors reached a mean of 1.555 days, while those treated with adjuvant therapy had a mean of 489 days (37, 40, 43). The data presented are described in Supplementary Table 8.

## 4. Discussion

The conventional treatment regimen for canine mast cell tumors is vinblastine (64), which is frequently combined with prednisolone (21, 36, 59). However, new therapies with good tolerance and clinical benefits are being evaluated for use, such as the use of TKI as molecular therapy, which is effective in humans in various types of malignancies, has been shown to be effective as a treatment for canine mast cell tumors (36). In this review, the overall survival of dogs treated with vinblastine alone was higher than the overall survival of dogs treated with TKI alone. This is intriguing since TKIs are important therapeutic resources capable of blocking cell signaling involved in mast cell tumor growth (65, 66), and were expected to have comparable efficiency to VBL. However, there are studies in the literature that demonstrate the existence of molecular mechanisms that provide resistance to TKIs, such as imatinib, during the treatment of neoplastic mast cells (66, 67), which would explain the higher survival rate in the VBL group. In addition to the cytotoxic action of VBL through binding to the microtubules inhibiting mitosis, another factor that may be associated with this result is the frequent association of VBL with prednisone, present in all studies in which the survival rate was analyzed. Prednisone is a glucocorticoid that can limit mast cell tumor proliferation *in vitro* and *in vivo*, decrease stem cell factor generation through fibroblasts and epithelial cells, and reduce inflammation (68–71). In studies evaluating the benefit of the combination of these two drugs, a higher OS and PFS were observed, demonstrating the efficacy of this treatment of canine mast cell tumors (59). A limitation of this systematic review was a lack of a prednisone + TKI group, which would have been useful to compare with the Other + VBL group.

The c-kit mutation test offers important prognostic information (8, 58). In this study, although dogs without the c-kit mutated had a higher OS compared to dogs that had the mutation, it was clear that the association of this mutation with TKI treatment prolonged the lives of these dogs when compared to those who received only vinblastine. This confirms that TKI has a high therapeutic effect in cases with c-kit mutation, and inhibition of the mutated form of c-kit can reduce the differentiation and survival of neoplastic mastocytes (53). In a more recent study, using vinblastine and toceranib, no positive correlation between the presence of the mutation and the response to treatment was observed, that is, the effectiveness of the drug was not altered by the mutation. However, the methodologies employed in this study were not sufficiently sensitive (72) and did not include prevalent mutations beyond exons 8 and 11. In addition, they have a conflict of interest with the pharmaceutical industry, which is responsible for producing toceranib, which could contribute to the unjustifiable use of TKIs (57). Therefore, dogs without the c-kit mutation could also show good results with TKI-based treatment. Moreover, the type of c-kit mutation present in the animal affects overall survival. Exon 8 ITD mutations in mast cell malignancies resulted in longer overall survival in dogs than exon 11 ITD mutations (73).

Despite these results indicated by the extracted data, it is important to note that all studies have performed conventional polymerase chain reaction (PCR)/sequencing methods for the detection of somatic mutations in canine mast cell samples. This can be considered a limitation since data indicate that this methodology has limited sensitivity (72, 74). In addition, all mutated tumors were based only on internal tandem duplications (ITD), and not on single-point mutations, and most performed sequencing only of exons 8 and 11, not providing information about other exons that may be constitutively activating, such as exon 9, 14, and 17 (12, 13, 75). Thus, due to the low sensitivity and allocation of groups of mutant and non-mutant dogs arbitrarily, it may be that dogs classified as non-mutant are mutated. It is recommended that studies be conducted with newer methodologies capable of detecting c-kit mutations with more sensitivity, such as COLD-PCR (72).

In the current study, progression-free survival of dogs that underwent vinblastine treatment alone was shown to be longer compared to dogs treated with TKI, according to data presented earlier, in which dogs treated with VBL had higher OS compared to dogs treated with TKI. This is possibly due to the association with prednisolone, which has already been associated with a high PFS. This contradicts other previous studies with inhibitors, in which they demonstrated longer progression-free survival for animals undergoing TKI treatment (57). This is thought-provoking, as for a new therapy to be approved for commercialization, it must meet unmet needs or ensure better outcomes than current therapies.

Other clinical parameters such as a good objective response rate and, consequently, lower adverse events could have influenced this survival (8, 59). In a study that evaluated groups of animals undergoing vinblastine-based treatment with other chemotherapeutics, they observed that patients undergoing this protocol had a longer disease-free time (49). In the current study, when the vinblastine group was analyzed with another chemotherapy agent, it was observed that there was a longer progression-free survival than the group of animals treated with inhibitors and another chemotherapy agent (8, 59, 76). However, the data were obtained only from a group of dogs submitted to palliative treatment, for this reason, the PFS was expected to present a lower value.

The median overall response rate, determined by tumor size or complete remission, varied according to the type of treatment, with a lower overall response rate observed when vinblastine was used alone compared to TKI treatment. This variability is possibly due to the type and aggressiveness of the mast cell tumor. Chemotherapy works mainly through cell ablation and tyrosine kinase inhibitors through growth limitation by inhibiting VEGFR2 and PDGFR, acting as an antiangiogenic agent and, as a result, causing tumor regression (77). Due to this, the contribution of TKI may be more beneficial in tumors with an aggressive and high proliferative profile. Although, the response rate is still an arbitrary endpoint result and in this systematic review not enough data was provided in each study to incontestably apply the overall response rate as a factor of better prognosis.

A contradiction was found in this study regarding the VBL group having better OS than the TKI group, and the TKI group had a greater clinical benefit of ORR compared to the VBL group. The relationship between response rate and survival is complex. Some authors suggest the lack of correlation between ORR and OS due to confounding factors, such as treatment crossing after progression, number of subsequent therapies, long post-progression survival in the first-line scenario, and non-cancer-related deaths (78). New targeted therapy agents may result in stabilization of the disease and not necessarily tumor regression, so response rates may be less valuable in accurately assessing biological activity and predicting the clinical benefit of the drug. Moreover, only predicting survival through tumor response goes beyond determining the number of responses, but also the duration of responses, the number of complete responses, and the location of responses (79).

A limitation of this study is related to sponsorship bias. In favorable results to the use of drugs, 28.5% (*n* = 6) were sponsored and 57% (*n* = 12) did not present any declaration of conflict of interest. Data indicate that studies funded by industries more often present data favorable to the use of the product produced compared to studies not sponsored by industry (odds ratio 4.05; 95%; confidence interval 2.98–5.51; 18 comparisons) (32, 80). Another point is that pharmaceutical companies can exercise limited power for unfavorable studies by withholding financial support (81).

Assuming this, the results should be interpreted with caution, and more studies should evaluate the clinical efficacy of these drugs without financial support from the industries, or even the provision of information on the status of sponsorship, which was absent in most studies.

It is important to emphasize that the data extracted from the studies did not present a sample standardization, that is, to obtain the general data, important characteristics about the patients, tumor, and type of treatment were not considered, which may interfere with the outcome of this study.

## 5. Conclusion

In conclusion, vinblastine had a greater clinical benefit than TKI in terms of overall survival and progression-free survival. However, TKI is more efficient in mast cell tumors with c-kit mutated. Although, without more robust results to assure greater efficacy in these patients, TKIs should not be considered a frontline for mast cell therapy.

## Data availability statement

The original contributions presented in the study are included in the article/Supplementary material, further inquiries can be directed to the corresponding author.

## Author contributions

YC performed the database searches and wrote the main manuscript text along with LS, PS, and CM. LP, TS, and MS were responsible for the process of searching and writing guidance of the manuscript. All authors contributed to the article and approved the submitted version.
