# Supplementary material for: Tyrosine kinase inhibitors as an alternative treatment in canine mast cell tumor
Source: Front Vet Sci. 2023 Jun 8;10:1188795. doi: 10.3389/fvets.2023.1188795 (PMC10285312; doi:10.3389/fvets.2023.1188795)
Supplement: Supplementary file 1 [file Data_Sheet_1.pdf]

Supplementary Material

Tyrosine kinase inhibitors as an alternative treatment in canine mast cell tumor

Yasmin Nascimento Bernardes Coelho[1]*, Luiz Ricardo Soldi[1], Paulo Henrique Rosa da Silva[1], Caio Melo Mesquita[2], Luiz Renato Paranhos[2], Thaísa Reis dos Santos[3] and Marcelo José Barbosa Silva[1]

*** Correspondence:** Corresponding Author: yasminnbcoelho@ufu.br

1 **Supplementary Table S1.** Studies excluded during full-text reading and the reasons for exclusion (n= 18).

Study excluded	Reason for exclusion
Halsey et al., 2007(1)	Study design: *in vitro* study.
Bailey et al., 2008(2)	Outcome: other variables.
Vickery et al., 2008(3)	Outcome: other variables.
Machicote, 2011(4)	Record not retrieved.
Chon et al., 2012(5)	Population: there were no dogs with mast cell tumor in the sample.
Justo, 2013(6)	Study design: literature review.
Halsey et al., 2014(7)	Study design: *in vitro* study.
Sousa, 2014(8)	Record not retrieved.
Krupa et al., 2015(9)	Record not retrieved.
Varela et al., 2016(10)	Outcome: other variables.
Bavcar et al., 2017(11)	Study design: case series with less than five individuals.
Halsey et al, 2017(12)	Study design: *in vitro* study.
Silva et al., 2017(13)	Study design: case series with less than five individuals.
Vincenti & Findji, 2017(14)	Outcome: other drugs.
Florindo, 2018(15)	Population: there were no dogs with mast cell tumor in the sample.
Souza et al., 2018(16)	Outcome: other variables.
Kluthcovsky et al., 2020(17)	Outcome: other variables.
Thamm et al., 2020(18)	Sample overlapping: same data as from an eligible study (Weishaar et al., 2017).

References

1. Halsey CHC. Markers and mechanisms of resistance to Toceranib phosphate (Palladia®) in canine cutaneous mast cell tumor. (2007) https://mountainscholar.org/bitstream/handle/10217/83771/Halsey_colostate_0053A_12367.pdf?sequence=1

2. Bailey DB, Rassnick KM, Kristal O, Chretin JD, Balkman CE. Phase I dose escalation of single-agent vinblastine in dogs. *J Vet Intern Med* (2008) 22:1397–1402. doi: 10.1111/J.1939-1676.2008.0196.X

3. Vickery KR, Wilson H, Vail DM, Thamm DH. Dose-escalating vinblastine for the treatment of canine mast cell tumour. *Vet Comp Oncol* (2008) 6:111–119. doi: 10.1111/J.1476-5829.2007.00147.X

4. Machicote G, Cobian A, Diaz-Santiago F. Treatment of canine mast cell tumors with a tyrosine kinase inhibitor. Regarding a clinical case. *Clin Vet PEQUENOS Anim* (2011) 31:19–27.

5. Chon E, Mccartan L, Kubicek LN, Vail DM. Safety evaluation of combination toceranib phosphate (Palladia®) and piroxicam in tumour-bearing dogs (excluding mast cell tumours): a phase I dose-

finding study. *Vet Comp Oncol* (2012) 10:184–193. doi: 10.1111/J.1476-5829.2011.00265.X

6. Justo BR de M. Avaliação da eficácia de protocolos terapêuticos para os mastocitomas caninos graus II e III. (2013)

7. Halsey CHC, Gustafson DL, Rose BJ, Wolf-Ringwall A, Burnett RC, Duval DL, et al. Development of an in vitro model of acquired resistance to toceranib phosphate (Palladia®) in canine mast cell tumor. *BMC Vet Res* (2014) 10: doi: 10.1186/1746-6148-10-105

8. Sousa OVV de. Aplicação do inibidor da tirosina cinase, fosfato de toceranib, no mastocitoma canino. (2014).

9. Krupa AM, De Vos JP, Polton G. Retrospective analysis of masitinib-based treatment of subcutaneous mast cell tumours in 25 chemo naïve dogs. *J Vet Intern Med* (2015) 29:456–457. https://www.embase.com/search/results?subaction=viewrecord&id=L623485573&from=export

10. Serra Varela JC, Pecceu E, Handel I, Lawrence J. Tolerability of a rapid-escalation vinblastine-prednisolone protocol in dogs with mast cell tumours. *Vet Med Sci* (2016) 2:266–280. doi: 10.1002/vms3.42

11. Bavcar S, de Vos J, Kessler M, de Fornel P, Buracco P, Murphy S, et al. Combination toceranib and lomustine shows frequent high grade toxicities when used for treatment of non-resectable or recurrent mast cell tumours in dogs: A European multicentre study. *Vet J* (2017) 224:1–6. doi: 10.1016/J.TVJL.2017.04.010

12. Halsey CHC, Thamm DH, Weishaar KM, Burton JH, Charles JB, Gustafson DL, et al. Expression of Phosphorylated KIT in Canine Mast Cell Tumor. *Vet Pathol* (2017) 54:387–394. doi: 10.1177/0300985816688943

13. Silva AFL. Mastocitomas cutâneos em cãe: análise dos protocolos de tratamento usado em cinco casos clínicos. (2017).

14. Vincenti S, Findji L. Influence of treatment on the outcome of dogs with incompletely excised grade-2 mast cell tumors. *Schweiz Arch Tierheilkd* (2017) 159:171–177. doi: 10.17236/SAT00109

15. Florindo APM. Estudo Retrospetivo Da Utilização Do Fosfato De Toceranib Numa Amostra De 38 Doentes Oncológicos Caninos. Universidade de Lisboa (Portugal) (2018).

16. Souza ACF, Pascoli AL, Ferreira M, Reis NP, Silva ICR, Santos RR, et al. Canine cutaneous mast cell tumors: retrospective study of cases attended by the oncology service at the Veterinary Hospital of FCAV-Unesp, Jaboticabal, from 2005 to 2015. *Pesqui Vet Bras* (2018) 39:1808–1817. doi: 10.1590/1678-5150-PVB-5150

17. Kluthcovsky LC, Firmo BF, Cassino PC, De Nardi AB, Castro JLC, Halila RL, et al. Comparison of two different vinblastine dosages for treatment of cutaneous mast cell tumor in dogs. *Acta Sci Vet* (2020) 48: doi: 10.22456/1679-9216.101997

18. Thamm DH, Weishaar KM, Charles JB, Ehrhart EJ. Phosphorylated KIT as a predictor of outcome in canine mast cell tumours treated with toceranib phosphate or vinblastine. *Vet Comp Oncol* (2020) 18:169–175. doi: 10.1111/vco.12525

Supplementary Table S2. Quantitative results of Overall Response Rate and Complete Response extracted from eligible studies.

Author, year	Groups (sample size) (n)	Results related to groups column*
	**Overall Response Rate**	
Thamm et al., 1999	VBL*<br>gross disease: 15	47% (n = 7)
David, et al, 2004	nr	nr
Thamm et al., 2006	nr	nr
Camps-Palauet et al., 2007	nr	nr
Hayes et al., 2007	nr	nr
Hanh et al., 2008	nr	nr
Isononi et al., 2008	TKI: 21	48% (n = 10)
Rassnick et al., 2008	VBL 2**: 26	27% (n = 7)
Rungsipipat, et al., 2008	nr	nr
Webster et al., 2008	nr	nr
Cooper et al., 2009	CCNU + VBL*: 56	56% (n = 31)
London et al., 2009	PCLB: 63<br>TKI: 86	PCLB: 7.9% (n = 5)<br>TKI: 37.2% (n = 32)
Hanh et al., 2010	nr	nr
Rassnick, et al., 2010	CCNU + VBL*: 52<br>nonresectable MCTs: 17	nonresectable MCTs: 65% (n = 11)
Carlsten, et al, 2012	TKI*: 17	76.4% (n = 13)
Robat et al., 2012	TKI + VBL: 14	71% (n = 10)
O'Connell, et al., 2013	nr	nr
Smrkovski et al., 2013	TKI: 26<br>first-line treatment: 14<br>second-line treatment: 12	first-line treatment: 50% (n = 7)<br>second-line treatment: 25% (n = 3)
Burton et al., 2015	TKI + CCNU: 41	46% (n = 19)
Lejuene et al., 2015	nr	nr
Chocteau, F. et al, 2016	nr	nr
Grant et al., 2016	TKI: 39	82.1% (n = 32)
Miller et al., 2016	nr	nr
Horta et al., 2018	TKI: 24	50% (n = 12)
Moirando et al., 2018	nr	nr
Olsen, et al., 2018	TKI + VBL: 29	measurable disease: 90% (n = 26)
Weishaar et al., 2018	VBL: 28<br>TKI: 60	VBL: 30% (n = 8)<br>TKI: 46 % (n = 28)
Todd et al., 2021	nr	nr
Macedo et al., 2022	VBL: 11<br>TKI: 13	VBL: 9.09% (n = 1)<br>TKI: 30.76% (n = 4)
	**Complete Response**	
Thamm et al., 1999	VBL*<br>gross disease: 15	33.33% (n = 5)
David, et al, 2004	VBL*: 20	70% (n = 14)
Thamm et al., 2006	nr	nr
Camps-Palauet et al., 2007	VBL* + CTX<br>measurable disease: 11	46% (n=5)
Hayes et al., 2007	nr	nr
Hanh et al., 2008	PCLB: 41<br>TKI: 161	PCLB: 21% (n = 9)<br>TKI: 26% (n = 45)
Isononi et al., 2008	TKI: 21	38.5% (n = 15)
Rassnick et al., 2008	VBL 2**: 26	4% (n = 1)
Rungsipipat, et al., 2008	VBL*: 23	21.8% (n = 5)
Webster et al., 2008	nr	nr
Cooper et al., 2009	CCNU + VBL*: 56<br>measurable disease: 37	measurable disease: 24% (n = 09)
London et al., 2009	PCLB: 63<br>TKI: 86	PCLB: nr<br>TKI: 8.4 % (n = 7)
Hanh et al., 2010	TKI: 67	9% (n = 6)
Rassnick, et al., 2010	CCNU + VBL*: 17	nonresectable MCTs: 29.4% (n = 5)
Carlsten, et al, 2012	TKI*: 17	58.8% (n = 10)
Robat et al., 2012	T + VBL: 14	14% (n = 2)
O'Connell, et al., 2013	nr	nr
Smrkovski et al., 2013	TKI: 26<br>first-line treatment: 14<br>second-line treatment: 12	first-line treatment: 27% (n = 3)<br>second-line treatment: nr
Burton et al., 2015	T + CCNU: 41	9.76% (n = 4)
Lejuene et al., 2015	nr	nr
Chocteau, F. et al, 2016	nr	nr
Grant et al., 2016	TKI: 39	82.1% (n = 32)
Miller et al., 2016	nr	nr
Horta et al., 2018	TKI: 24	29% (n =7)

Moirando et al., 2018	nr	nr
Olsen, et al., 2018	TKI + VBL: 40	Neoadjuvant: 38% (6/16)<br>Adjuvant: 45% (5/11)<br>Palliative: 92% (12/13)
Weishaar et al., 2018	VBL: 28<br>TKI: 60	VBL: 4 (n =1)<br>TKI: 13% (n = 7)
Todd et al., 2021	nr	nr
Macedo et al., 2022	VBL: 11<br>TKI: 13	VBL: nr<br>TKI: 7.69% (n =1)

nr – not reported in the study; * percentage of the values displayed in the left column (n); TKI – Tyrosine Kinase Inhibitors; VBL – Vinblastine; VBL* – Vinblastine in combination with prednisone; VBL 2**: VBL at a dosage of 3.5 mg/m²; T – Toceranib; M – Masitinib; I – Imatinib; PCB – placebo; TKI* - associated with hypofractionated radiation treatment and prednisolone; CTX-Cyclophosphamide; CCNU – Lomustine.

3 **Supplementary Table S3.** Quantitative results of Partial Response extracted from eligible studies.

Author, year	Groups (sample size) (n)	Results related to groups column*
**Partial Response**		
Thamm et al., 1999	VBL*<br>gross disease: 15	13.33% (n = 2)
David, et al, 2004	nr	nr
Thamm et al., 2006	nr	nr
Camps-Palauet et al., 2007	VBL* + CTX<br>measurable disease: 11	18% (n = 2)
Hayes et al., 2007	nr	nr
Hanh et al., 2008	PCLB: 41<br>TKI: 161	PCLB: 36% (n = 15)<br>TKI: 55% (n = 91)
Isotoni et al., 2008	TKI: 21	19% (n = 4)
Rassnick et al., 2008	VBL 2**: 26	23% (n = 6)
Rungsipipat, et al., 2008	VBL*: 23	78.2% (n = 18)
Webster et al., 2008	nr	nr
Cooper et al., 2009	CCNU + VBL*: 56<br>measurable disease: 37	measurable disease: 32% (n=12)
London et al., 2009	PCLB: 63<br>TKI: 86	PCLB: 3.36% (n = 5)<br>TKI: 16.7% (n = 25)
Hanh et al., 2010	nr	nr
Rassnick, et al., 2010	CCNU + VBL*: 17	nonresectable MCTs: 35.2% (n = 6)
Carlsten, et al, 2012	TKI*: 17	17.6% (n = 3)
Robat et al., 2012	T + VBL: 14	8.57% (n =1)
O'Connell, et al., 2013	nr	nr
Smrkovski et al., 2013	TKI: 26<br>first-line treatment: 14<br>second-line treatment: 12	first-line treatment: 15% (n = 2)
Burton et al., 2015	T + CCNU: 41	36.59% (n = 15)
Lejuene et al., 2015	nr	nr
Chocteau, F. et al, 2016	nr	nr
Grant et al., 2016	TKI: 39	43.6% (n = 17)
Miller et al., 2016	nr	nr
Horta et al., 2018	TKI: 24	21% (n = 5)
Moirando et al., 2018	nr	nr
Olsen, et al., 2018	nr	nr
Weishaar et al., 2018	nr	nr
Todd et al., 2021	nr	nr
Macedo et al., 2022	VBL: 11<br>TKI: 13	VBL: 9.09% (n = 1)<br>TKI: 23.7% (n = 3)

nr – not reported in the study; * percentage of the values displayed in the left column (n); TKI – Tyrosine Kinase Inhibitors; VBL – Vinblastine; VBL* – Vinblastine in combination with prednisone; VBL 2**: VBL at a dosage of 3.5 mg/m²; T – Toceranib; M – Masitinib; I – Imatinib; PCB – placebo; TKI* - associated with hypofractionated radiation treatment and prednisolone; CTX-Cyclophosphamide; CCNU – Lomustine.

Supplementary Table S4. Quantitative results of Overall Survival extracted from eligible studies.

	Overall Survival	
Thamm et al., 1999	VBL*	grade III MCT: 331 days
David, et al, 2004	nr	nr
Thamm et al., 2006	VBL*	1374 days
Camps-Palauet et al., 2007	VBL* + CTX	1118.5 days<br>measurable disease: 145 days<br>incompletely excised MCT: 2092 days
Hayes et al., 2007	nr	nr
Hanh et al., 2008	TKI/PCB	TKI:<br>491 days<br>mutated kit: 417 days<br>PCB:<br>340 days<br>mutated kit: 242 days
Isotoni et al., 2008	nr	nr
Rassnick et al., 2008	nr	nr
Rungsipipat, et al., 2008	VBL*	101 days
Webster et al., 2008	VBL*	440.33 days<br>grade II: 654.41 days<br>grade III: 280 days<br>kit II: 578 days<br>kit III: 328 days<br>mutated kit: 270 days<br>wild – type kit: 529 days
Cooper et al., 2009	CCNU + VBL*	209.5 days<br>measurable disease: 245 days<br>incompletely excised MCT: 336 days
London et al., 2009	T/PCB	TKI: 168 days<br>PCB: nr
Hanh et al., 2010	TKI/PCB	TKI: 617 days<br>PCB: 322 days
Rassnick, et al., 2010	nr	nr
Carlsten, et al, 2012	nr	nr
Robat et al., 2012	nr	nr
O'Connell, et al., 2013	TKI/VBL	TKI: 182.5 days<br>VBL: 167.2 days
Smrkovski et al., 2013	nr	nr
Burton et al., 2015	TKI + CCNU	nr
Lejuene et al., 2015	VBL* + CCNU	1359 days
Chocteau **et** al, 2016	TKI	high-grade: 432 days<br>low-grade: nr<br>mutated kit: 505 days<br>wild – type kit: 1389 days
Grant et al., 2016	TKI	159 days
Miller et al., 2016	TKI/VBL*	TKI<br>323.5 days<br>grade II: 369 days<br>grade III: 278 days<br>VBL<br>1018.2 days<br>grade II: 1946 days<br>grade III: 91 days
Horta et al., 2018	TKI	113 days
Moirando et al., 2018	TKI/VBL	TKI: 118 days<br>VBL: 227 days
Olsen, et al., 2018	TKI + VBL	neoadjuvant: nr<br>adjuvant: 893 days<br>palliative: 218 days
Weishaar et al., 2018	TKI/VBL	TKI: 159 days<br>VBL: 241.5 days
Todd et al., 2021	TKI + VBL	high- grade: 563 days
Macedo et al., 2022	nr	nr

nr – not reported in the study; * percentage of the values displayed in the left column (n); TKI – Tyrosine Kinase Inhibitors; VBL – Vinblastine; VBL* – Vinblastine in combination with prednisone; VBL 2**: VBL at a dosage of 3.5 mg/m²; T – Toceranib; M – Masitinib; I – Imatinib; PCB – placebo; TKI* - associated with hypofractionated radiation treatment and prednisolone; CTX- Cyclophosphamide; CCNU – Lomustine.

5 **Supplementary Table S5.** Quantitative results of Progression-Free Survival extracted from eligible studies**.**

**Progression-Free Survival**		
Thamm et al., 1999	VBL*	nr
David, et al, 2004	nr	nr
Thamm et al., 2006	VBL*	1305 days
Camps-Palauet et al., 2007	VBL* + CTX	608 days<br>measurable disease: 74 days<br>incompletely excised MCT: 2865 days
Hayes et al., 2007	VBL*	491 days
Hanh et al., 2008	TKI/PCB	TKI:<br>118 days<br>first line: 253 days<br>as adjuvant: 84 days<br>mutated kit: 230 days<br>wild–type kit: 72 days<br>PCB:<br>mutated kit: 83 days<br>wild–type kit: 66 days
Isotoni et al., 2008	nr	nr
Rassnick et al., 2008	nr	nr
Rungsipipat, et al., 2008	nr	nr
Webster et al., 2008	TKI	429.17 days
Cooper et al., 2009	VBL* + CCNU	227.5 days<br>measurable disease: 210 days<br>incompletely excised MCT: 245 days
London et al., 2003	T/PCB	nr
Hanh et al., 2011	nr	nr
Rassnick, et al., 2010	VBL* + CCNU	adjuvant setting: 489 days.<br>grade III: 190 days<br>metastatic grade II: 954 days
Carlsten, et al, 2012	TKI*	316 days<br>mutated kit: 188.5 days<br>wild – type kit: nr
Robat et al., 2012	nr	nr
O'Connell, et al., 2013	TKI/VBL	TKI: 30 days<br>VBL: 45 days
Burton et al., 2015	TKI + CCNU	53 days
Lejuene et al., 2015	VBL* + CCNU	2120 days
Smrkovski et al., 2013	TKI	first-line treatment: 453 days
Chocteau, F. et al, 2016	nr	nr
Grant et al., 2016	TKI	79 days
Miller et al., 2016	nr	nr
Horta et al., 2018	TKI	140 days
Moirando et al., 2018	nr	nr
Olsen, et al., 2018	TKI + VBL	neoadjuvant: nr<br>adjuvant: nr<br>palliative: 45 days
Weishaar et al., 2018	TKI/VBL	TKI: 95.5 days<br>VBL: 78 days
Todd et al., 2021	TKI + VBL	high- grade: 398 days
Macedo et al., 2022	nr	nr

nr – not reported in the study; TKI – Tyrosine Kinase Inhibitors; VBL – Vinblastine; VBL* – Vinblastine in combination with prednisone; VBL 2**: VBL at a dosage of 3.5 mg/m²; T – Toceranib; M – Masitinib; I – Imatinib; PCB – placebo; TKI* - associated with hypofractionated radiation treatment and prednisolone; CTX- Cyclophosphamide; CCNU – Lomustine.

6. Supplementary Table S6. Main methods of mutation detection in studies that showed c-kit mutation status.

Authors, year	Detection methods
**Hanh et al., 2008**	The RNA was isolated and extracted and transcribed through reverse transcriptase and complementary DNA (cDNA) was obtained, which was amplified by PCR with the primers covering exons 8–13 and 17–19. The amplifiers were purified Amplimers with the GeneClean III kit and directly sequenced with the Big Dye Terminator V 1.1 kit and sequencing primers on an ABI Prism 3130 sequencer. Observed mutations were systematically checked in a new reverse transcription reaction. This method was able to detect the mutated allele when present in 5–10% of the cells.
**Isotoni et al., 2008**	Genomic DNA was extracted from tumor cells collected by needle aspiration or from surgically excised tumor tissues. The RNAs were reverse transcribed into cDNA with reverse transcriptase. Aliquot cDNA was submitted to PCR amplification with a set of primers to amplify the exon 11 c-kit. After amplification, products were fractionated by size in a 2% agarose gel and visualized with ethidium bromide staining. A band of PCR products larger than the estimated size (190 bp) of the wild-type c-kit amplification product was extracted from the gel and nucleotide sequences were determined.
**London et al., 2009**	Samples were taken from the tumor, skin adjacent to the tumor and normal skin at a distant site of the tumor through a puncture biopsy instrument. The samples were placed in individual boxes microcentrifuge tubes and digested overnight at 37°C in a standard proteinase K buffer. The samples were quantified and were then used PCR to detect ITDs present in the juxtamembrane domain of the c-kit. The forward primer for this reaction was placed at the 5' end of exon 11, and the reverse primer was placed at the 5' end of intron 11. PCR products were analyzed by agarose gel electrophoresis (4%); tumors possessing tandem duplications inc-kit generated both a wild-type PCR product as well as a larger product representing that containing the tandem duplication. Controls for these studies included the genomic DNA derived from the C2 canine mastocytoma cell line (containing an ITD and no wild-type Kit) and canine cerebellum.
**Hanh et al., 2010**<br>**Carlsten, et al, 2012**<br>**Burton et al., 2015**	The presence of mutations in the sequence of the c-Kit receptor expressed by the tumors was evaluated by reverse transcriptase-PCR assay performed on biopsy specimens. Genomic DNA samples were prepared from fine needle aspirates stained with Wright-Giemsa or samples fixed in formalin and embedded in paraffin. The slides were confirmed to have 10% of the cell population as mast cells. Qiagen AL buffer was applied to the blades, which were then scraped with a straight-tipped razor into microcentrifuge tubes. DNA extraction was then performed with a commercial kit according to the manufacturer's instructions. Regions of c-kit exons 8 and 11 were amplified with primers directed against sequences flanking the characterized ITDs (internal tan-dem duplications). The amplifications were performed with a commercially available PCR kit. Genetic scanning analysis was performed using a capillary electrophoresis machine. Raw data were analyzed with commercially available genotype analysis software. Genomic DNA samples were prepared from fine needle aspirates stained with Wright-Giemsa or samples fixed in formalin and embedded in paraffin. The slides were confirmed to have 10% of the cell population as mast cells. Qiagen AL buffer was applied to the blades, which were then scraped with a straight-tipped razor into microcentrifuge tubes. DNA extraction was then performed with a commercial kit according to the manufacturer's instructions. Regions of c-kit exons 8 and 11 were amplified with primers directed against sequences flanking the characterized ITDs (internal tandem duplications). The amplifications were performed with a commercially available PCR kit. Genetic scanning analysis was performed using a capillary electrophoresis machine. Raw data were analyzed with commercially available genotype analysis software.
**Horta et al., 2018**	The polymerase chain reaction (PCR) for amplification of the fragment of interest in the c-kit oncogene, was performed by Progen, in Vetpat Laboratory (Campinas, SP, Brazil), from the DNA extraction in paraffin embedded tumour, by the proteinase K method. The primers used in the bleaching of the reaction were designed with the help of the BLAST software (Basic Local Alignment Search Tool®, NCBI) and manufactured by Invitrogen, as c-kit forward: 5'-ATCTGT CTC TCT TTT CTC CCC C-3' (sense) and c-kit reverse: 5' -TGG GGT TCC CTA AAG TCA TTG T-3' (antisense). The product generated by these pair of primers had 225 bp in the absence of mutations (native c-kit). Reactions were prepared and planned in a GenPro thermocycler (BIOER Technology), with a maintenance at 95℃ for five min, then 30 cycles of 94℃ for 45 sec for denaturation of DNA strands, 63℃ for 45 sec to pairing and annealing of primers and 72℃ for one minute to extension, to be finally maintained at 72℃ for ten min for molecular stabilization. The amplified material was separated by electrophoresis at 100V, with free amperage. Canine healthy skin samples and milique water were used as positive and negative controls, respectively. Genetic assessment of exon 11 was performed using PCR analysis rather than genetic sequencing, so point mutations in exon 11, could not be assessed. Primers applied in this study were limited only to exon 11, but there might be c-kit activating mutations in other loci, like exons 2, 5, 6, 7, 8, 9 and 1.
**Weishaar et al., 2018**	The needle-aspirated samples were submitted to PCR-forced mutation kit detection. Internal tandem duplications in exon 8 and exon 11 were detected using primers designed to amplify the areas of reported mutation. Reported DNA was amplified in a thermocycler by the following protocol: 94° for 5 minutes, 95° for 15 minutes, 60° for 1 minute, 40 cycles. The PCR products were analyzed in a capillary electrophoresis machine through the Proteomics and Metabolomics Facility. During the development of this assay, it was determined that at least 10% of the cells in the preparation must be mast cells for detection of the kit-c mutation. Therefore, the samples were inspected for cell composition before use.

**Macedo et al., 2022**	The polymerase chain reaction (PCR) for amplification of the fragment of interest in the c-kit oncogene, was performed by Progen, in Vetpat Laboratory (Campinas, SP, Brazil), from the DNA extraction in paraffin embedded tumour, by the proteinase K method. The primers used in the bleaching of the reaction were designed with the help of the BLAST software (Basic Local Alignment Search Tool®, NCBI) and manufactured by Invitrogen, as c-kit forward: 5'-ATCTGT CTC TCT TTT CTC CCC C-3' (sense) and c-kit reverse: 5' -TGG GGT TCC CTA AAG TCA TTG T-3' (antisense). The product generated by these pair of primers had 225 bp in the absence of mutations (native c-kit). Reactions were prepared and planned in a GenPro thermocycler (BIOER Technology), with a maintenance at 95℃ for five min, then 30 cycles of 94℃ for 45 sec for denaturation of DNA strands, 63℃ for 45 sec to pairing and annealing of primers and 72℃ for one minute to extension, to be finally maintained at 72℃ for ten min for molecular stabilization. The amplified material was separated by electrophoresis at 100V, with free amperage. Canine healthy skin samples and milique water were used as positive and negative controls, respectively. Genetic assessment of exon 11 was performed using PCR analysis rather than genetic sequencing, so point mutations in exon 11, could not be assessed. Primers applied in this study were limited only to exon 11, but there might be c-kit activating mutations in other loci, like exons 2, 5, 6, 7, 8, 9 and 1.

RNA - ribonucleic acid; DNA - deoxyribonucleic acid; cDNA - complementary DNA; PCR - polymerase chain reaction; ITD - internal tandem duplications; MCT – mast cell tumor.

7. **Supplementary Table S7.** Mean overall, complete, and partial responses extracted from selected publications in the four groups.

**Overall Response Rate**			
**Author, year**	**N total**	**N**	**%***
**TKI**			
London, et al., 2003	86	32	37.2%
Isononi et al., 2008	21	10	48%
Carlsten, et al, 2012	17	13	76.4%
Smrkovski et al., 2015	14	7	50%
Grant et al., 2016	39	32	82.1%
Horta et al., 2018	24	12	50%
Weishaar et al., 2018	60	28	46%
Macedo et al., 2022	13	4	30.76%
**TOTAL**	**274**	**138**	**50.36%**
**VBL**			
Thamm et al., 1999	15	7	47%
Rassnick et al., 2008	26	7	27%
Smrkovski et al., 2015	12	3	25%
Weishaar et al., 2018	28	8	30%
Macedo et al., 2022	11	1	9.09%
**TOTAL**	**92**	**26**	**28.26%**
**Chemotherapy+TKI**			
Robat et al., 2012	14	10	71%
Burton et al., 2015	41	19	46%
Olsen, et al., 2018	29	26	90%
**TOTAL**	**84**	**55**	**65.47%**
**Others+VBL**			
Cooper et al., 2009	56	31	57%
Rassnick, et al., 2010	17	11	65%
**TOTAL**	**73**	**42**	**57.53%**
**Complete Response**			
**Author, year**	**N total**	**N**	**%**
**TKI**			
London, et al., 2003	86	7	8.4%
Hanh et al., 2008	161	45	26%
Isononi et al., 2008	21	15	38.5%
Hanh et al., 2010	67	6	9.0%
Carlsten, et al, 2012	17	10	58.8%
Smrkovski et al., 2015	14	3	27%
Grant et al., 2016	39	32	82.1%
Horta et al., 2018	24	7	29%
Weishaar et al., 2018	60	7	13%
Macedo et al., 2022	13	1	7.69%
**TOTAL**	**502**	**133**	**26.49%**
**VBL**			
Thamm et al., 1999	15	5	33.3%
David, et al, 2004	20	14	70%
Rassnick et al., 2008	26	1	4.0%
Rungsipipat, et al., 2008	23	5	21.8%

Weishaar et al., 2018	28	1	4.0%
**TOTAL**	**112**	**26**	**23.21%**
		**Chemotherapy+TKI**	
Robat et al., 2012	14	2	14%
Burton et al., 2015	41	4	9.76%
Olsen, et al., 2018	40	26	65%
**TOTAL**	**95**	**32**	**33.68%**
		**Others+VBL**	
Camps-Palauet et al., 2007	11	5	46%
Cooper et al., 2009	37	9	24%
Rassnick, et al., 2010	17	5	29.4%
**TOTAL**	**65**	**19**	**29.23%**
		**Partial Response**	
**Author, year**	**N total**	**N**	**%**
		**TKI**	
London, et al., 2003	86	25	16.7%
Hanh et al., 2008	161	91	55%
Isotoni et al., 2008	21	4	19%
Carlsten, et al, 2012	17	3	17.6%
Smrkovski et al., 2015	14	2	15%
Grant et al., 2016	39	17	43.6%
Horta et al., 2018	24	5	21%
Macedo et al., 2022	13	1	7.69%
**TOTAL**	**375**	**148**	**39.46%**
		**VBL**	
Thamm et al., 1999	15	2	13.33%
Rassnick et al., 2008	26	6	23%
Rungsipipat, et al., 2008	23	18	78.2%
Macedo et al., 2022	11	1	9.09%
**TOTAL**	**75**	**27**	**36%**
		**Chemotherapy+TKI**	
Robat et al., 2012	14	1	8.57%
Burton et al., 2015	41	15	36.59%
**TOTAL**	**55**	**16**	**29.1%**
		**Others+VBL**	
Camps-Palauet et al., 2007	11	2	18%
Cooper et al., 2009	37	12	32%
Rassnick, et al., 2010	17	6	35.2%
**TOTAL**	**65**	**20**	**30.7%**

8. **Supplementary Table S8**. Survival time and Progression-Free Survival extracted from selected publications in the four groups.

Author, year	ALL PATIENTS	Grade II	Grade III	Mutated Kit	Not Mutated Kit
**TKI**					
London, et al., 2003	168 days	nr	nr	nr	nr
Hanh, et al., 2008	491 days	nr	nr	nr	nr
Hanh, et al., 2010	617 days	nr	nr	417 days	nr
O'Connell, et al., 2013	182 days	nr	nr	nr	nr
Chocteau, et al, 2016	775 days	nr	nr	505 days	1389 days
Grant et al., 2016	152 days	nr	nr	nr	nr
Miller et al., 2016	323.5 days*	369 days	278 days	nr	nr
Horta et al., 2018	113 days	nr	nr	nr	nr
Moirando et al., 2018	118 days	nr	nr	nr	nr
Weishaar et al., 2018	159 days	nr	nr	nr	nr
**MEAN**	**308.3 days**	**369 days**	**278 days**	**461 days**	**1389 days**
**VBL**					
Thamm et al., 1999;	331 days	nr	331 days	nr	nr
Thamm et al., 2006	1374 days	nr	nr	nr	nr
Webster et al., 2008	440.33 days	654.41 days	280.02 days	270.89 days	529.61 days
Rungsipipat, et al., 2010	101 days	nr	nr	nr	nr
O'Connell, et al., 2013	167.2 days	nr	nr	nr	nr
Miller et al., 2016	1018.2 days	1946 days	91 days	nr	nr
Weishaar et al., 2017	241.5 days	nr	nr	nr	nr
**MEAN**	**524.7 days**	**1300 days**	**234 days**	**270.89 days**	**529.61 days**
**Chemotherapy+TKI**					
Olsen, et al., 2018	555.5 days**	nr	nr	nr	nr
Todd et al., 2021	563 days	nr	nr	nr	nr
**MEAN**	**559.25 days**	**nr**	**nr**	**nr**	**nr**
**Others+VBL**					
Camps-Palauet et al., 2007	1118.5 days	nr	nr	nr	nr
Cooper et al., 2009	209.5 days	nr	nr	nr	nr
Lejuene et al., 2015	1359 days	nr	nr	nr	nr
Moirando et al., 2017	227 days	nr	nr	nr	nr
**MEAN**	**728.5 days**	**nr**	**nr**	**nr**	**nr**
**PROGRESSION-FREE SURVIVAL**					
**Author, year**	**ALL PATIENTS**	**Grade II**	**Grade III**	**Mutated Kit**	**Not Mutated Kit**
**TKI**					
Hanh et al., 2008	118 days	nr	nr	230 days	72 days
Webster et al., 2008	429.17 days	nr	nr	nr	nr
Carlsten, et al, 2012	316 days	nr	nr	188.5 days	nr
O'Connell, et al., 2013	30 days	nr	nr	nr	nr
Smrkovski et al., 2015	453 days	nr	nr	nr	nr
Grant et al., 2016	79 days	nr	nr	nr	nr
Horta et al., 2018	140 days	nr	nr	nr	nr
Weishaar et al., 2018	95.5 days	nr	nr	nr	nr
**MEAN**	**207.5 days**	**nr**	**nr**	**209.2 days**	**72 days**
**VBL**					
Thamm et al., 2006	1305 days	nr	nr	nr	nr
Hayes et al., 2007	491 days	nr	nr	nr	nr
O'Connell, et al., 2013	45 days	nr	nr	nr	nr
Weishaar et al., 2018	1018.2 days	nr	nr	nr	nr
**MEAN**	**714.8 days**	**nr**	**nr**	**nr**	**nr**
**Chemotherapy+TKI**					
Burton et al., 2015	53 days	nr	nr	nr	nr
Olsen, et al., 2018	45 days***	nr	nr	nr	nr
**MEAN**	**49 days**	**nr**	**nr**	**nr**	**nr**
**Others+VBL**					
Camps-Palauet et al., 2007	608 days	nr	nr	nr	nr
Cooper et al., 2009	227.5 days	nr	nr	nr	nr
Rassnick, et al., 2010	489 days	954 days	190 days	nr	nr
Lejuene et al., 2015	2120 days	nr	nr	nr	nr
**MEAN**	**861.1 days**	**954 days**	**190 days**	**nr**	**nr**

nr – not reported in the study; *Mean days performed with dogs with histological grade II and III to obtain the total mean; **Mean days obtained from dogs palliative and adjuvant treatment to obtain total mean; ***Data obtained only from dogs undergoing palliative treatment.
